# Malignant Melanoma of the Female Genital Tract: Experience of an Oncology Center in Pakistan

**DOI:** 10.7759/cureus.8484

**Published:** 2020-06-07

**Authors:** Osama Shakeel, Faizan Ullah, Nazish Khalid, Shalla I Ali, Sadaf Batool, Awais Amjad, Abdul Wahid Anwer, Hannan Ali, Hania Zafar, Aamir Ali Syed

**Affiliations:** 1 Surgical Oncology, Shaukat Khanum Memorial Cancer Hospital and Research Centre, Lahore, PAK; 2 Surgical Oncology, Shaukat Khanum Memorial Cancer Hospital and Research Center, Karachi, PAK; 3 Surgical Oncology, Shaukat Khanum Memorial Cancer Hospital and Research Center, Lahore, PAK; 4 Surgery, Shaukat Khanum Memorial Cancer Hospital and Research Centre, Lahore, PAK; 5 Surgery, Shaukat Khanum Memorial Cancer Hospital and Research Center, Lahore, PAK; 6 Obstetrics and Gynecology, The Indus Hospital, Lahore, PAK; 7 Obstetrics and Gynecology, Nishtar Medical College and Hospital, Multan, PAK

**Keywords:** rare cancers of female genital tract, malignant melanoma, lower middle income country, mucosal malignant melanoma, malignant melanoma of female genital tract

## Abstract

Introduction

Malignant melanoma, which arises from melanocytes or pigment cells, is one of the most common forms of epithelial cancer. Cutaneous and noncutaneous melanomas differ clinically and genetically. Mucosal melanomas are rare. In the female genital tract, the most frequent location of melanoma is the vulva, whereas the vagina is seldom affected. The occult nature of their anatomical location contributes to the late presentation and late diagnosis of vaginal melanoma, resulting in an exceedingly poor patient prognosis. The present study describes the incidence, symptoms, management, and prognosis of women in Pakistan with malignant melanoma of the vulva, vagina, and cervix.

Materials and methods

The Hospital Information System of Shaukat Khanam Memorial Cancer Hospital and Research Center was searched electronically to identify patients diagnosed with malignant melanoma from January 1995 to December 2017. Patients with cutaneous malignant melanoma, multiple primary tumors, and metastases to the female genital tract from primary tumors located elsewhere were excluded. All included patients had been diagnosed with primary malignant melanoma of the female genital tract.

Results

The search of medical records identified 271 patients with malignant melanoma, of whom 13 had primary malignant melanomas of the female genital tract. Of these 13 patients, nine, three, and one had primary vaginal, vulvar, and cervical melanomas, respectively. Median age at presentation was 60 years (range, 30-70 years), with 10 patients being post-menopausal. The most common presentations were per-vaginal bleeding and per-vaginal discharge (five patients each). The mean duration of symptoms was 7.46 months. Seven patients underwent wide local excision. Six patients had nodular type malignant melanoma, two had superficial spreading type, and five were unclassified. Nine patients had pathological T4 disease, and two had pathological T3. Mean Breslow depth was 5.4 millimeters (mm), with 10 patients having tumor depth >4 mm. Eight patients were positive for the microscopic involvement of margins. The mean time to recurrence was 11.8 months (range, 1-24 months), and the mean time to metastasis was 17.6 months (range, 2-44 months). The median survival after surgery was 25 months (range, 2-75 months).

Conclusion

This study is the first to report the incidence, symptoms, management, and prognosis of patients in Pakistan with malignant melanoma of the female genital tract. Meta-analyses and prospective multicenter studies are needed.

## Introduction

Malignant melanoma, which arises from melanocytes or pigment cells, is one of the most common types of epithelial cancer, diagnosed in 76,000 people in the United States annually [1]. Most melanocytes are present in skin, but they are also found in various mucosal linings, including those of the gastrointestinal, respiratory, and urogenital tracts [2]. Malignant melanomas can be broadly divided into two groups, mucosal (noncutaneous) and nonmucosal (cutaneous), which differ clinically and genetically [3,4]. Cutaneous melanomas are the sixth most common type of cancer among women in the United States, whereas mucosal melanomas are rare and account for only 2% of all melanomas [5,6].

Gender plays an important role in the epidemiology of mucosal melanomas. As its incidence is about two-fold higher in women than in men, mucosal melanomas are significantly more often diagnosed in women [5,7]. This gender gap is due to the disparity in the incidence of genital tract mucosal melanomas, which is higher in women, whereas the incidence of extra-genital mucosal melanomas is about the same in both sexes. The most frequent location of melanoma in the female genital tract is the vulva, whereas the vagina is seldom affected, with the annual incidence of primary melanoma of the vagina (PMV) being about three per 10,000,000 women [5]. In contrast, primary melanomas of the vulva are four to nine times more frequent than PMV and account for three-fourths of all genital melanomas [8,9]. PMV usually occurs in older women and is most frequently diagnosed at an advanced stage, resulting in early recurrence and a poor prognosis [5]. The five-year overall survival (OS) rate in patients with PMV is about 18%, compared with five-year OS rates of 47% in patients with vulvar melanoma and 81% in patients with cutaneous melanoma [5,7,9,10]. The occult nature of their anatomical location contributes to the late presentation and late diagnosis of PMV. In addition, the diffuse lymphatic vascular plexus in the vagina promotes early metastasis of vaginal melanoma.

Because of their rarity, there are currently no established guidelines for the treatment of genital melanomas. Complete resection may be difficult due to their anatomical location, and vaginal melanomas are often resistant to chemotherapy and radiotherapy [11]. The present study describes the incidence, symptoms, management, and prognosis of women in Pakistan with malignant melanoma of the vulva, vagina, and cervix.

## Materials and methods

The Hospital Information System of Shaukat Khanam Memorial Cancer Hospital and Research Center (SKMCH&RC) was searched electronically to identify patients diagnosed with malignant melanoma from January 1995 to December 2017. Patients with cutaneous malignant melanoma, multiple primary tumors, and metastases to the female genital tract from primary tumors located elsewhere were excluded. All included patients had been diagnosed with primary malignant melanoma of the female genital tract. The study protocol was approved by the Institutional Review Board of SKMCH&RC, which waived the requirement for informed consent due to the retrospective design of the study.

Factors recorded included times from diagnosis to surgery, from surgery to recurrence, and from surgery to metastasis. The histopathologic characteristics of the tumors were evaluated, including the type of malignant melanoma and Breslow depth. Outcomes evaluated included age, menopausal status, management, OS, disease-free survival (DFS), recurrence, and site of metastasis.

All statistical analyses were performed using Statistical Packages for Social Sciences (SPSS) for Windows, Version 22.0 (IBM Corp., Armonk, NY)). Non-normally distributed quantitative variables were reported as median with minimum and maximum, whereas categorical variables were reported as numbers and percentages. OS and DFS were calculated using the Kaplan-Meyer method.

## Results

A search of the electronic medical records of SKMCH&RC identified 271 patients diagnosed with malignant melanoma over the 23-year study period. Of these patients, 174 (64.2%) had primary cutaneous, and 97 (35.8%) had primary noncutaneous malignant melanoma. Of the 97 patients with primary noncutaneous malignant melanoma, 89 (91.8%) had mucosal, five (5.2%) had ocular, and three (3.1%) had visceral melanoma. Thirteen patients were diagnosed with primary female genital malignant melanoma; of these, nine, three, and one were diagnosed with vaginal, vulvar, and cervical melanoma, respectively.

At the time of presentation, the median age of the 13 women diagnosed with primary genital malignant melanoma was 60 years (range, 30-70 years), with 10 patients being post-menopausal at presentation. The most common presentations were per-vaginal bleeding and per-vaginal discharge (five patients each), followed by vaginal growth (two patients) and pain (one patient). The mean duration of symptoms was 7.46 months, although eight patients consulted a physician within four months of symptom onset (Table *1*).

**Table 1:** Clinicopathological characteristics of the 13 women with malignant melanoma of the genital tract Tis, carcinoma in situ

Patient No.	Age (years)	Menopausal status	Site of tumor	Breslow thickness (mm)	Tumor stage	Nodal status	M stage	Symptoms	Symptom duration (months)
1	50	Post	Vulva	10	T4	N0	M0	Vaginal discharge	5
2	30	Pre	Vulva	0.75	Tis	N0	M0	Swelling	2
3	63	Post	Vulva	8	T4	N2	M0	Vaginal bleeding	3
4	65	Post	Vagina	4	T4	N2	M0	Vaginal discharge	36
5	32	Pre	Vagina	4	T4	Nx	M0	Vaginal discharge	4
6	30	Pre	Vagina	4	T4	N0	M1	Vaginal bleeding	2
7	70	Post	Vagina	3	T3,	No	M0	Vaginal discharge	2
8	60	Post	Vagina	4	T4	Nx	M0	Pain	4
9	63	Post	Vagina	3.5	T2	Nx	M0	Vaginal bleeding	12
10	48	Post	Vagina	10	T4	N0	M0	Vaginal discharge	3
11	50	Post	Vagina	4	T3	No	M0	Vaginal bleeding	8
12	60	Post	Vagina	9	T4	Nx	M0	Vaginal growth	12
13	68	Post	Cervix	6	T4	Nx	M0	Vaginal bleeding	4

Of the 13 patients, seven underwent wide local excision, whereas two underwent radical resection, one undergoing total abdominal hysterectomy and the other undergoing bilateral salpingo-oophorectomy, vulvectomy, and sub-total vaginectomy. The other two patients were found to have distant metastases at the time of presentation and were offered palliative therapy, but one of these patients refused palliative treatment and was lost to follow-up.

Six patients had nodular type malignant melanoma, two had a superficial spreading type, and five were unclassified. Nine patients had advanced pathological T4 disease, two had pathological T3, and one each had pathological T2 and carcinoma in situ. Malignant melanoma is widely classified into thin, intermediate, and deep thickness. Mean Breslow depth was 5.4 millimeter (mm), with 10 patients having a depth >4 mm, two having a depth of 0.76-4 mm, and one having a depth <0.76 mm thin thickness. Eight patients were positive for the microscopic involvement of the margins.

Only one patient with distant metastasis received palliative radiotherapy. Seven patients received adjuvant radiotherapy, whereas one patient received both chemotherapy and radiotherapy. Five patients developed tumor recurrence during follow-up, with three having a local recurrence, one having a recurrence at regional lymph nodes and one having a recurrence at both local and regional lymph nodes. Of these five patients, only two had margin-positive disease on initial biopsy. In addition, distant metastasis occurred in nine patients, with four having lung and three having both lung and liver metastases simultaneously. One patient had brain metastasis, and one developed distant nodal dissemination. The median time to recurrence was 11 months (range, 1-24 months), and the median time to metastasis was 17 months (range, 2-44 months). The median OS after surgery was 25 months (range, 2-75 months).

## Discussion

Although malignant melanoma is not uncommon, genital melanoma is a rare subtype with a reported incidence of 0.46 per million women worldwide [12]. The present study found that only 13 women who presented at SKMCH&RC over 17 years had genital melanoma. It is considered a disease of the elderly, as it rarely occurs in premenopausal women [13]. The mean age at the time of presentation was reported to be 57 years, consistent with our finding that the median age of these 13 women was 60 years [14]. Their rarity and the occult nature of their location result in the late presentation and delayed diagnosis of these tumors.

Similarly, our patients presented after a mean symptom duration of seven months. Although the vulva has been reported to be the most frequently affected area of the female genital tract, we found that the vagina was the most frequent site of the melanoma. Vaginal nodularity is the most common presentation among western women, whereas we found that per-vaginal bleeding and discharge were the most frequent symptoms at presentation [15,16].

Traditionally malignant melanoma staging depends on Breslow thickness and tumor-node-metastasis (TNM) stages. The National Comprehensive Cancer Network provides detailed guidelines for the treatment of patients with cutaneous malignant melanoma [17]. Mohs micrographic surgery is recommended for melanoma in situ and Lantigo maligna. In contrast, wide local excision, sentinel lymph node biopsy with or without complete dissection of the nodal basin, and combination treatment with BRAF inhibitors have been recommended for patients with more advanced tumors [17,18]. Among these BRAF inhibitors are the anti-BRAF monoclonal antibodies vemurafenib and dabrafenib [19]. Unlike cutaneous malignant melanomas, the prevalence of BRAF mutations in mucosal malignant melanoma is usually accompanied by c-kit mutations [20].

Currently, there are no consensus guidelines for the treatment of genital malignant melanoma. Acceptable options are wide local excision, radical excision, chemotherapy, chemoradiotherapy, immunotherapy, and their various combinations. Most of our patients underwent wide local excision or radical excision, followed by adjuvant therapy. Of our 13 patients, eight had positive margins, which were likely due to early loco-regional tumor spread and the presence of a diffuse lymphatic vascular plexus in the female genital tract [10].

Irrespective of treatment modality, prognosis in patients with these tumors is generally dismal, with no difference in survival between radical and wide local excision [8,21]. The five-year OS rate is less than 10%, whereas the five-year OS rate of women with vulvar melanoma is slightly better, ranging from 15% to 54% [22,23]. These rates have not changed since the early 1980s, with the five-year OS rate after radical vulvectomy, inguinal lymph node dissection, and adjuvant radiotherapy being 31.6% [24].

The infrequent incidence of these tumors is the main hindrance in developing treatment protocols. Widely accepted treatment options for these tumors include surgical excision followed by adjuvant chemotherapy, chemoradiotherapy, or immunotherapy. Routine gynecological examinations should include a thorough examination of the genitalia, especially in older women.

The limitations of this study include its retrospective nature and small sample size. Due to the considerable time involved, the quality of data retrieved was not up to the standards required for a detailed discussion of this disease. To our knowledge, this study is the first from South Asia to evaluate the clinicopathological factors, management, and survival of women with genital malignant melanoma disease. Meta-analyses and prospective multicenter studies are required to confirm these findings.

## Conclusions

Genital melanoma is a rare disease with poor patient prognosis. Complete resection may be difficult due to their anatomical location, and vaginal melanomas are often resistant to chemotherapy and radiotherapy. This disease is associated with poor prognosis. The goal of the study is to raise awareness regarding the knowledge of the clinicopathological characteristics and survival outcomes of genital malignant melanoma from the Pakistani cohort. Although surgery and chemotherapy are regarded as essential to its management, the rarity of this disease has hindered efforts to develop management guidelines. Our findings will be used as a reference point for future studies.
